# TAB-TICI Score: Successful Recanalization Score After Endovascular Thrombectomy in Acute Stroke

**DOI:** 10.3389/fneur.2021.692490

**Published:** 2021-10-14

**Authors:** Woo-Keun Seo, Hyo Suk Nam, Jong-Won Chung, Young Dae Kim, Keon-Ha Kim, Oh Young Bang, Byung Moon Kim, Gyeung-Moon Kim, Pyoung Jeon, Ji Hoe Heo

**Affiliations:** ^1^Department of Neurology and Stroke Center, Samsung Medical Center, Sungkyunkwan University School of Medicine, Seoul, South Korea; ^2^Department of Digital Health, Samsung Advanced Institute for Health Science & Technology (SAIHST), Sungkyunkwan University, Seoul, South Korea; ^3^Department of Neurology, Yonsei University College of Medicine, Seoul, South Korea; ^4^Department of Radiology, Samsung Medical Center, School of Medicine, Sungkyunkwan University, Seoul, South Korea; ^5^Interventional Neuroradiology, Yonsei University College of Medicine, Seoul, South Korea

**Keywords:** endovascular thrombectomy, outcome, performance, stroke, recanalization

## Abstract

**Background and Purpose:** Successful reperfusion therapy is supposed to be comprehensive and validated beyond the grade of recanalization. This study aimed to develop a novel scoring system for defining the successful recanalization after endovascular thrombectomy.

**Methods:** We analyzed the data of consecutive acute stroke patients who were eligible to undergo reperfusion therapy within 24 h of onset and who underwent mechanical thrombectomy using a nationwide multicenter stroke registry. A new score was produced using the predictors which were directly linked to the procedure to evaluate the performance of the thrombectomy procedure.

**Results:** In total, 446 patients in the training population and 222 patients in the validation population were analyzed. From the potential components of the score, four items were selected: Emergency Room-to-puncture time (T), adjuvant devices used (A), procedural intracranial bleeding (B), and post-thrombectomy reperfusion status [Thrombolysis in Cerebral Infarction (TICI)]. Using these items, the TAB-TICI score was developed, which showed good performance in terms of discriminating early neurological aggravation [AUC 0.73, 95% confidence interval (CI) 0.67–0.78, *P* < 0.01] and favorable outcomes (AUC 0.69, 95% CI 0.64–0.75, *P* < 0.01) in the training population. The stability of the TAB-TICI score was confirmed by external validation and sensitivity analyses. The TAB-TICI score and its derived grade of successful recanalization were significantly associated with the volume of thrombectomy cases at each site and in each admission year.

**Conclusion:** The TAB-TICI score is a valid and easy-to-use tool to more comprehensively define successful recanalization after endovascular thrombectomy in acute stroke patients with large vessel occlusion.

## Introduction

Endovascular thrombectomy (EVT) with substantial recanalization is a crucial determinant of the functional independence of acute stroke patients with large vessel occlusion (LVO) (1). The likelihood of achieving functional independence increases with improved recanalization in patients treated with intravenous thrombolysis (2) or EVT (3, 4). Therefore, to obtain the maximum benefit, current guidelines recommend achieving modified Thrombolysis in Cerebral Infarction (mTICI) 2b or 3 grade angiographic results as a technical goal of EVT (1). This substantial reperfusion is often regarded as a successful recanalization therapy. However, the status of reperfusion alone is insufficient as a criterion for a successful procedure because approximately three-quarters of the patients undergo EVT to obtain substantial reperfusion (mTICI 2b or 3) (5) and a great proportion of patients with substantial reperfusion show poor functional outcomes (6). Moreover, performance of procedures in patients with failed reperfusion should be further stratified, suggesting the need for a comprehensive evaluation tool for successful thrombectomy. However, except for angiographic reperfusion, there is no validated tool to evaluate the successful recanalization.

We, therefore, developed a novel scoring system to assess successful recanalization and tested its performance using an external dataset. Additionally, we validated the successful recanalization scoring system with different parameters in a nationwide multicenter cohort.

## Methods

### Study Design and Data Source

We analyzed data from the SElection CRiteria in Endovascular Thrombectomy and Thrombolytic Therapy registry (Clinicaltrials.gov NCT02964052). A total of 17 sites across South Korea were included in this project. Eligibility criteria were as follows: (1) age ≥18 years; (2) initial National Institutes of Health Stroke Scale (NIHSS) score ≥4; (3) direct admission to each participating hospital without hospital-to-hospital transfer; (4) onset-to-puncture time ≤600 min; (5) modified Rankin Score (mRS) before the qualifying stroke of 0/1; (6) recanalization success assessable on catheter angiogram; (7) documented catheter-accessible cerebral artery occlusion (intracranial internal carotid artery, middle cerebral artery M1 or proximal M2 occlusion, anterior cerebral artery, posterior cerebral artery, basilar artery or vertebral artery) on computed tomography, magnetic resonance imaging (MRI), or catheter angiography; and (8) available functional outcomes (mRS = 0–6) at 3 months ± 2 weeks. Because the primary purpose of this study was to build a model for measuring the performance of thrombectomy, patients who did not undergo thrombectomy were excluded (7). This project constituted two parts: a retrospective part that analyzed pre-existing prospective registries from each site from 2012 to 2015 and a prospective registry from November 2016 to December 2017.

The protocol of the entire project was approved by the ethical review board of each participating site. For patients enrolled retrospectively, informed consent was waived by the institutional review board because of the retrospective design. All patients who participated in the prospective part of the project provided informed consent.

### Data Collection and Assessment

Each participating site entered the data for demographic characteristics, vascular risk factors, neurological status, time metrics for reperfusion therapy, procedural details, and functional outcomes using a web-based case report form. All imaging data were sent to the central laboratory in an anonymized DICOM format. Reperfusion grade was independently assessed by two neuroradiologists according to the mTICI scale, and decisions were made by consensus in case of discrepancies. Early neurological aggravation was defined as any increase in the NIHSS score at 24 h from baseline. A favorable outcome was defined as an mRS of 0–2 at 90 days.

### Predictive Model Construction, Validation, and Statistical Analysis

The primary purpose of this study was to produce a scale for evaluating successful recanalization. For this purpose, we established three principles: (1) the items in the score should be confined to those directly associated with thrombectomy (several well-known outcome predictors were omitted if the items could not be manipulated by the interventional operators or acute stroke management team); (2) the scoring system should be valid for evaluating functional neurological outcomes and risk of procedure-related neurological worsening; and (3) the scale should be related to the metrics with a known association with successful recanalization, such as the volume of thrombectomy at each site (7) or on each admission date.

We divided the dataset into training (retrospective) and validation (prospective) datasets and used only the training dataset for producing the score. All available variables in the database were screened and selected when the items were potentially associated with favorable outcomes or early neurological aggravation. Especially, the adjuvant device use was defined when the second thrombectomy device system replaced the first thrombectomy device system. To construct a predictive model for outcomes using the selected items, we performed univariate and multivariate logistic regression analyses with each item and selected items that were significantly associated with either outcome. Item selection and score rating were performed according to the probability obtained with a logistic model incorporating receiver operating curve (ROC) analyses. On the basis of the results of these analyses, we developed a new score.

The stability of the developed score was internally validated with *k*-fold cross-validation using the area under the curve (AUC). The model was tested using prospectively collected validation datasets and presented with sensitivity, specificity, and AUC.

We divided the population into three groups according to performance: excellent, fair, and poor. In addition, we validated the clinical implications of the TAB-TICI score in terms of volume of cases at each site and in each admission year as well as changes in trends with each admission year. The independent associations among these clinical implications were proved using ordinal logit regression analysis with covariates.

All statistical analyses were performed using STATA (STATA version 15, StataCorp LLC, USA), and *P* < 0.05 was considered significant.

## Results

### Study Population

From 1359 patients registered in the cohort from 17 sites, 653 (45.2% females; mean age, 69.2 ± 12.3 years) patients who were treated with intravenous thrombolysis alone and 38 patients who underwent catheter-based angiography without thrombectomy were finally excluded in this study; 691 patients were excluded. Finally, there were 446 patients (47.1% females; mean age, 69.3 ± 12.3 years) in the training population (retrospective part) and 222 patients (41.4% females; mean age, 69.1 ± 12.5 years) in the validation population (prospective part) (Table 1).

**Table 1:** Baseline characteristics of the training and validation populations.

**Characteristic**	**Training population (*n* = 446)**	**Validation population (*n* = 222)**	***P*-value**
Age (years), mean ± SD	69.3 ± 12.3	69.1 ± 12.5	0.99
Sex, female, *n* (%)	210 (47.1)	92 (41.4)	0.17
Hypertension, *n* (%)	332 (74.4)	157 (70.7)	0.31
Diabetes mellitus, *n* (%)	209 (46.9)	98 (44.1)	0.51
Hypercholesterolemia, *n* (%)	164 (36.8)	90 (40.5)	0.35
Current smoking, *n* (%)	82 (18.4)	37 (16.7)	0.58
Coronary artery disease *n* (%)	104 (23.3)	37 (16.7)	0.047
Atrial fibrillation, *n* (%)	259 (58.1)	108 (48.6)	0.021
Congestive heart failure, *n* (%)	40 (9.0)	12 (5.4)	0.11
Early neurological aggravation, *n* (%)^*^	87 (19.5)	31 (14.0)	0.08
Favorable outcome, *n* (%)^†^	180 (45.5)	99 (58.9)	<0.01
Onset-to-ER time, median (IQR)	120 (50.8–268.3)	88 (40–266)	0.22
Puncture-to-reperfusion time, median (IQR)	36 (25–47)	33 (22–44)	0.03
Puncture-to-reperfusion time >35 min, *n* (%)	242 (54.3)	105 (47.3)	0.09
ER-to-puncture time, median (IQR)	116 (93–147)	100 (80–128.5)	<0.01
ER-to-puncture time >110 min, *n* (%)	210 (52.6)	85 (41.5)	<0.01
First-pass recanalization, *n* (%)	162 (36.3)	90 (40.5)	0.289
Number of thrombectomy pass, median (IQR)	2 (1–4)	2 (1–3)	<0.01
Adjuvant device (number)			<0.01
None	351 (78.7)	211 (95)	
One adjuvant device	86 (19.3)	11 (5.0)	
Second or more adjuvant device	9 (2.0)	0 (0.0)	
Adjuvant device (class)			
Second stent retriever, *n* (%)	2 (0.4)	1 (0.5)	0.997
Stent retriever + contact aspiration, *n* (%)	67 (15)	0 (0)	<0.01
Intracranial stent or angioplasty, *n* (%)	37 (8.3)	10 (4.5)	0.07
Adjuvant chemical thrombolysis, *n* (%)	202 (45.3)	93 (41.9)	0.41
Carotid stent, *n* (%)	34 (7.6)	20 (9.0)	0.54
Non-bleeding procedural complication, *n* (%)	30 (6.7)	25 (11.3)	0.045
Procedural intracranial bleeding, *n* (%)	36 (8.1)	23 (10.4)	0.33
Subarachnoid hemorrhage, *n* (%)	11 (2.5)	6 (2.7)	0.86
Intracerebral hemorrhage, *n* (%)	21 (4.7)	17 (7.7)	0.12
Intraventricular hemorrhage, *n* (%)	12 (2.7)	1 (0.5)	0.048
mTICI, *n* (%)			0.01
0 or 1	57 (12.8)	13 (5.9)	
2a	44 (9.9)	18 (8.2)	
2b or 3	344 (77.3)	189 (85.9)	
Target vessel, *n* (%)			0.67
Internal carotid artery	147 (33.6)	66 (30.4)	
Middle cerebral artery (M1)	187 (42.8)	94 (43.3)	
Middle cerebral artery (M2)	39 (8.9)	24 (11.1)	
Other anterior circulation	2 (0.5)	1 (0.5)	
Posterior circulation	62 (14.2)	31 (14.3)	
NIHSS	15.4 ± 5.9	13.7 ± 6.3	<0.01

* *Early neurological aggravation was defined any increase in NIHSS at 24 h from baseline*.

† *Favorable outcome was defined as Modified Rankin Score of 0–2 at 90 days*.

MCA M1 was the leading site of occlusion (42.1%), followed by the internal carotid artery (31.9%), posterior circulation (13.9%), and MCA M2 (9.4%). Mean NIHSS score was 14.9 ± 6.1 [median 15, interquartile range (IQR) 11–19]. Median (IQR) time between onset and hospital visit was 113 (49–268) min. Mechanical thrombectomy produced a substantial recanalization, as defined by mTICI grades 2b-3, in 80.2% of patients. Favorable outcomes and early neurological aggravation were reported in 49.5 and 17.7% of patients, respectively.

Thrombectomy was performed using a retrievable stent in most cases (83.8%), and a contact aspiration device was used as the first or adjuvant device in 19%. During the procedure, 8.8% of patients experienced procedure-related intracranial bleeding, and 8.2% suffered from procedural adverse events other than intracranial bleeding.

### Model Development

Using univariate analysis, we selected the major categories of each item for developing the new score (Table 2). Final items were selected using multivariate analyses (Table 3). Because the model was required to predict both early neurological aggravation and favorable outcomes at 90 days, all items from the models for early neurological aggravation and favorable outcome were selected. Finally, ER-to-puncture time (T), adjuvant devices used (A), procedural intracranial bleeding (B), and post-thrombectomy reperfusion status (TICI) were selected as items for the final model.

**Table 2:** The association of each predictor with early neurological aggravation and the favorable outcome.

	**Early neurological aggravation**		**Favorable outcome**	
	**OR (95% CI)**	***P*-value**	**OR (95% CI)**	***P*-value**
ER-to-puncture >110 min	1.80 (1.08–3.00)	0.03	0.58 (0.38–0.88)	0.01
Puncture-to-recanalization >35 min	0.70 (0.44–1.12)	0.14	1.47 (0.99–2.20)	0.06
First-pass recanalization	0.80 (0.48–1.31)	0.37	1.46 (0.97–2.20)	0.07
Adjuvant device (yes or no)	3.35 (2.01–5.59)	<0.01	0.36 (0.21–0.62)	<0.01
Second stent retriever	4.16 (0.26–67.22)	0.32	NA	
Stent retriever plus aspiration	3.31 (1.88–5.80)	<0.01	0.26 (0.13–0.50)	<0.01
Additional angioplasty or stenting	2.45 (1.19–5.04)	0.02	0.68 (0.31–1.46)	0.32
Number of adjuvant devices				
0	1		1	
1	3.24 (1.91–5.50)	<0.01	0.38 (0.22–0.67)	<0.01
≥2	4.60 (1.20–17.70)	0.03	0.17 (0.02–1.38)	0.10
Adjuvant chemical thrombolysis	1.64 (1.02–2.63)	0.04	0.96 (0.65–1.43)	0.85
Carotid stent	1.30 (0.57–2.97)	0.54	1.89 (0.88–4.04)	0.10
Non-procedural adverse events	0.82 (0.30–2.19)	0.69	0.96 (0.44–2.10)	0.91
Procedural intracranial bleeding	2.23 (1.07–4.67)	0.03	0.19 (0.07–0.51)	<0.01
Subarachnoid hemorrhage	5.24 (1.56–17.61)	<0.01	–	
Intracerebral hemorrhage	0.97 (0.32–2.96)	0.96	0.21 (0.06–0.74)	0.02
Intraventricular hemorrhage	6.20 (1.92–20.02)	<0.01	0.13 (0.02–1.02)	0.05
SAH or IVH	4.49 (1.73–11.67)	<0.01	0.08 (0.01–0.62)	0.02
Procedural intracranial bleeding				
None	1		1	
ICH only	–		0.24 (0.05–1.14)	0.07
SAH or IVH	4.35 (1.67–11.31)	<0.01	0.08 (0.01–0.60)	0.01
mTICI				
0 or 1	1		1	
2a	0.15 (0.06–0.42)	<0.01	1.46 (0.51–4.17)	0.48
2b or 3	0.17 (0.10–0.31)	<0.01	6.54 (2.98–14.37)	<0.01

*SAH, subarachnoid hemorrhage; IVH, intraventricular hemorrhage; ICH, intracerebral hemorrhage*.

**Table 3:** Selection of items from the major categories related to early neurological aggravation and favorable outcomes.

	**Early neurological aggravation**				**Favorable outcome**			
	**Univariate**		Multivariate^*^		**Univariate**		Multivariable^*^	
	**OR (95% CI)**	***P*-value**	**OR (95% CI)**	***P*-value**	**OR (95% CI)**	***P*-value**	**OR (95% CI)**	***P*-value**
ER-to-puncture >110 min	1.80 (1.08–3.00)	0.03	1.73 (1.00–2.97)	0.048	0.58 (0.38–0.88)	0.01		
Puncture-to-recanalization >35 min	1.43 (0.89–2.28)	0.14			1.47 (0.99–2.20)	0.06		
Presence of adjuvant device	3.35 (2.01–5.59)	<0.01	3.40 (1.91–6.04)	<0.01	0.36 (0.21–0.62)	<0.01	0.49 (0.26–0.90)	0.02
Adjuvant chemical thrombolysis	1.64 (1.02–2.63)	0.04			0.96 (0.65–1.43)	0.85		
Non-procedural adverse events	0.82 (0.30–2.19)	0.69			0.96 (0.44–2.10)	0.91		
Procedural intracranial bleeding	2.23 (1.07–4.67)	0.03			0.19 (0.07–0.51)	0.01	0.24 (0.09–0.66)	0.01
Failed thrombectomy (mTICI 0–2a)	2.98 (1.80–4.9)	<0.01	2.01 (1.14–3.55)	0.02	0.18 (0.10–0.32)	<0.01	0.25 (0.14–0.45)	<0.01

* *Multivariate logistic model was the final model using backward conditional covariate selection*.

ROC analyses to find the best model using differently weighted items showed that the model with a 0–2 range for reperfusion status score showed the highest AUC for both early neurological aggravation and favorable outcomes (Supplementary Table 1). Finally, we developed a single score ranging 0–5 and including four items (TAB-TICI score; Table 4).

**Table 4:** Components of the TAB-TICI score.

**Acronym**	**Item**	**Response**	Score^*^
T	ER-to-puncture time	>110 min	1
		≤110 min	0
A	Adjuvant thrombectomy device^†^	Yes	1
		No	0
B	Procedure-related intracranial bleeding^‡^	Yes	1
		No	0
TICI	mTICI	0 or 1	2
		2a	1
		2b or 3	0
TAB-TICI			0–5

* *The TAB-TICI score was calculated as T score + A score + B score + TICI score*.

† *The class of adjuvant devices included: (1) a second stent retriever, (2) a stent retriever plus contact aspiration device, and (3) a stent retriever/contact aspiration device plus angioplasty and/or stenting*.

‡ *Intracranial bleeding includes any intracerebral hemorrhage, subarachnoid hemorrhage, or intraventricular hemorrhage*.

### Internal Validation, External Validation, and Sensitivity Analyses of the TAB-TICI Score

The TAB-TICI score showed fair-to-good performance in discriminating early neurological aggravation [AUC 0.73, 95% confidence interval (CI) 0.67–0.78, *P* < 0.01] and favorable outcomes (AUC 0.69, 95% CI 0.64–0.75, *P* < 0.01) in the training population (Table 5).

**Table 5:** Internal and external validation of the TAB-TICI score performance.

	**Sensitivity**	Specificity^*^	**AUC (95% CI)**	***P*-value**
Training population				
Early neurological aggravation (*n* = 398)	53.8%^*^	78.4%^*^	0.73 (0.67–0.78)	<0.01
Favorable outcome (*n* = 352)	84.8%^*^	40.6%^*^	0.69 (0.64–0.75)	<0.01
*k*-fold cross-validation				
Early neurological aggravation (*n* = 398)	76.9%^‡^	49.1%^‡^	0.72 (0.60–0.74)	
Favorable outcome (*n* = 352)	72.4%^†^	48.7%^†^	0.70 (0.61–0.73)	
Validation population				
Early neurological aggravation (*n* = 204)	50.0%^*^	83.7%^*^	0.68 (0.60–0.79)	<0.01
Favorable outcome (*n* = 157)	93.5%^*^	37.5%^*^	0.66 (0.57–0.75)	<0.01

* *The study population was classified into two groups: TAB-TICI score 0–2 and TAB-TICI score 3–5*.

*For calculating the sensitivity, specificity, and accuracy, the TAB-TICI score 0–2 group was considered positive for favorable outcomes, whereas the TAB-TICI score 3–5 group was considered positive for early neurological aggravation*.

† *At a predicted probability of 45.6%*.

‡ *At a predicted probability of 19.5%*.

When the TAB-TICI score was dichotomized into TAB-TICI 0–2 and TAB-TICI 3–5, the sensitivity and specificity were 53.8 and 78.4%, respectively, for early neurological aggravation and 84.8 and 40.6%, respectively, for favorable outcomes. *k*-fold cross-validation (internal validation) revealed similar results (AUC 0.72, 95% CI 0.60–0.74 for early neurological aggravation; AUC 0.70, 95% CI 0.61–0.73 for favorable outcomes; Supplementary Figure 1; Table 3). External validation for the validation population also revealed a similar trend (AUC 0.68, 95% CI 0.60–79, *P* < 0.01 for early neurological aggravation; AUC 0.66, 95% CI 0.57–0.75, *P* < 0.01 for favorable outcomes; Table 5).

**Figure 1:**
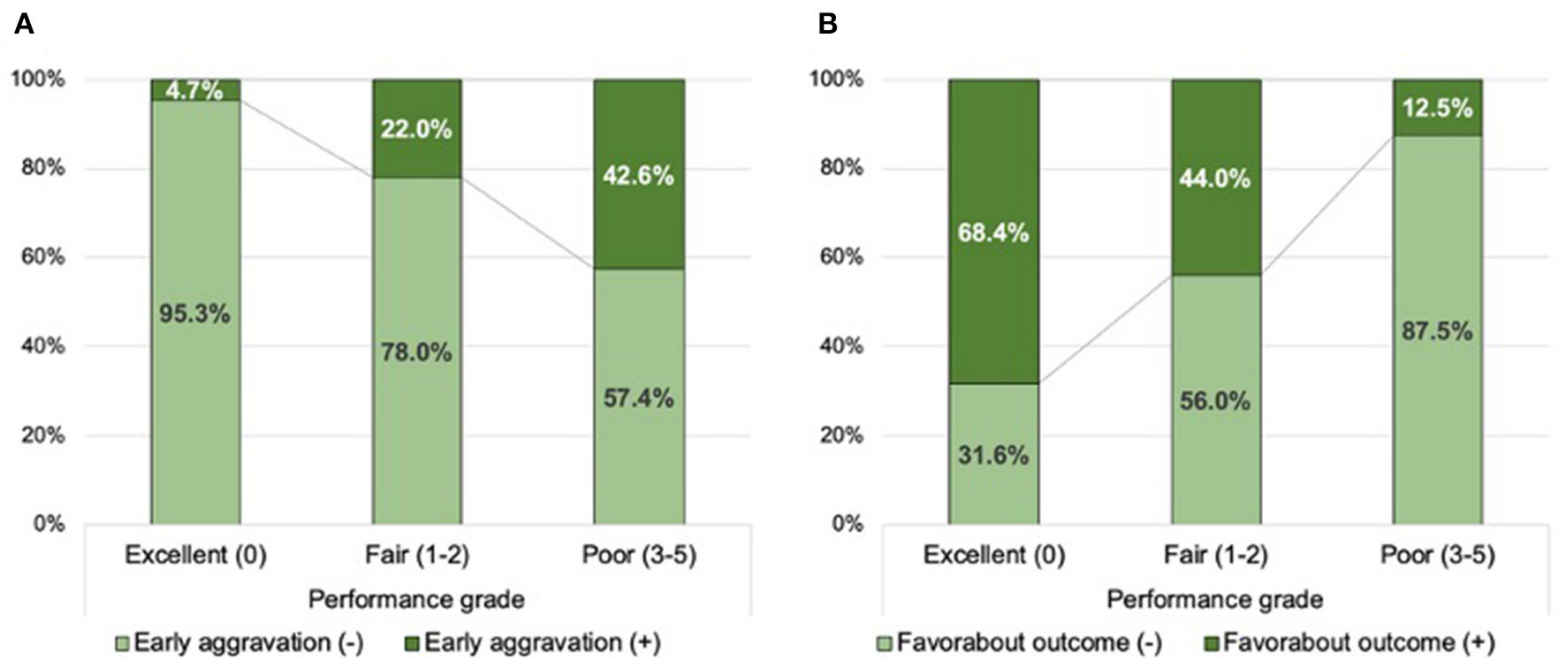
Distribution of the successful recanalization score according to early neurological aggravation **(A)** and favorable outcomes **(B)** in the training population.

Supplementary Figure 2 demonstrates the association between the TAB-TICI score and accumulated proportions of outcomes. On the basis of these results, we classified the performance into three grades: excellent (TAB-TICI score 0), fair (TAB-TICI scores 1 and 2), and poor (TAB-TICI scores 3–5). The proportion of early neurological aggravation sequentially increased (*P*-value for Kendall tau <0.01) and that of favorable outcomes decreased (*P*-value for Kendall tau <0.01; Figure 1 with worsening of performance grades from excellent to poor.

Sensitivity analyses were performed to demonstrate stability of the TAB-TICI score. The TAB-TICI score maintained the association with early neurological aggravation (AUC 0.67, 95% CI 0.60–0.74, *P* = 0.04) and favorable outcomes (AUC 0.63, 95% CI 0.57–0.68, *P* = 0.03) in patients with substantial reperfusion (mTICI 2b or 3). Moreover, there was no significant difference between anterior (AUC 0.74, 95% CI 0.68–0.80, *P* = 0.01 for early neurological aggravation; AUC 0.70, 95% CI 0.65–0.75, *P* < 0.01 for favorable outcome) and posterior (AUC 0.69, 95% CI 0.56–0.81, *P* = 0.01 for early neurological aggravation; AUC 0.71, 95% CI 0.59–0.83, *P* < 0.01 for favorable outcomes) circulation stroke patients.

### Association of Performance Grade With Thrombectomy Case Volume at Each Participating Site and in Each Admission Year

**Figure 2:**
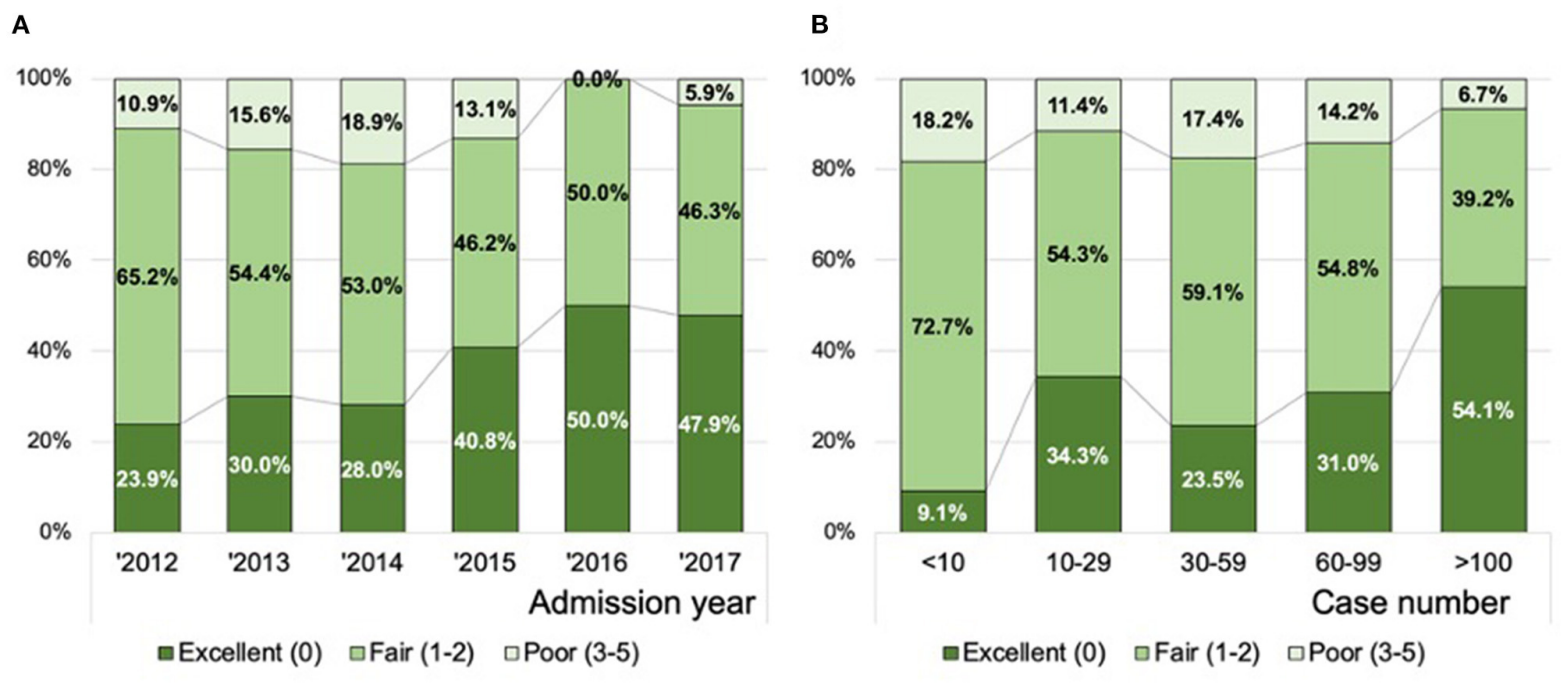
Distribution of the successful recanalization score according to the year of admission **(A)** and the volume of thrombectomy cases at each site **(B)**. Data in combined training and all validation populations are presented (*n* = 602).

In ordinal logistic regression analyses, the TAB-TICI score was negatively associated with the volume of thrombectomy cases at each site [adjusted odds ratio (aOR) 0.992, 95% CI 0.990–0.994, *P* < 0.01] and decreased over time (aOR 0.76, 95% CI 0.69–0.84, *P* < 0.01). Figure 2 demonstrates the association of performance grade (excellent, good, and poor) with admission year (Figure 2A) and the volume of thrombectomy cases at each site (Figure 2B). The proportion of excellent performance was 23.9% in 2012, which increased gradually and became 47.9% in 2017 (*P*-value for Kendall tau <0.01). The volume of thrombectomy cases at each site was also significantly associated with the performance grade, with a higher proportion of excellent performance observed in the group with a higher volume of thrombectomy cases (*P*-value for Kendall tau <0.01).

## Discussion

In this study, using a nationwide multicenter registry for acute stroke patients who underwent EVT, we developed a new scoring system to assess the successful recanalization of endovascular thrombectomy comprising four items: ER-to-puncture time, adjuvant devices used, procedural intracranial bleeding, and post-thrombectomy reperfusion status. Using these items, we developed the TAB-TICI score (with a higher score indicating a worse performance) and tested its internal and external validity. Decreased TAB-TICI score or shift from poor to excellent performance grade derived from the TAB-TICI score was associated with an increased volume of thrombectomy cases at each site and in later admission years. This association reflects the feasibility of using the TAB-TICI score as a metric for evaluating EVT performance.

We selected four items to develop the new score on the basis of favorable functional outcomes and risk of early neurological aggravation. Reperfusion, not simply angiographic recanalization, was the most critical predictor of EVT outcome, and this finding is consistent with those of prior investigations (8, 9). However, further stratification according to the risk of outcome events is required. In this regard, the TAB-TICI score exhibited potential usefulness in stratifying the performance of patients with or without substantial reperfusion in sensitivity analysis.

Workflow time metrics are well-known predictors of outcomes (10) and are the primary targets for quality assurance and control in acute stroke management at the same time. In this study, we selected the interval from emergency room (ER) arrival to groin puncture on the basis of analytical results. Previous studies support the importance of ER-to-puncture time (11, 12). Saver and colleagues addressed the relative importance of ER-to-puncture time over onset-to-puncture time using analysis of the Highly Effective Reperfusion Using Multiple Endovascular Devices study (11). They suggested that the vague definition of symptom onset time and the possibility for eliminating the “onset-to-ER effect” through deselection of fast or very slow progressors in the clinical trials have resulted in the observed trend. The importance of the ER-to-puncture time effect was doubled by the fact that the rate of achievement of substantial reperfusion decreased with an increase in ER-to-groin puncture time, although this was not associated with the total onset-to-puncture time (12). Additionally, because the collaboration of multiple domains and parties in a hospital is essential to effectively reduce the workflow time metrics in an acute stroke management setting, ER-to-puncture time as an item related to procedural performance is advantageous as it represents the capability of the acute stroke care team.

We considered another time metric—the puncture-to-reperfusion time—as an item related to procedural performance. However, we discarded it because puncture-to-reperfusion time was not associated with early neurological aggravation and the significance of its association with favorable outcomes in univariate analysis was eliminated in multivariate analysis.

In terms of technical aspects of thrombectomy, we screened for the following items as a measure of performance: a number of passes for stent retrieval or aspiration (13), first-pass recanalization (14), adjuvant chemical thrombolysis (15), and adjuvant intracranial angioplasty or stenting in cases of intracranial atherosclerotic disease (16). Among these, only adjuvant thrombectomy device use was selected as an item in the developed scoring system via covariate selection using a multivariate logistic regression model. In contrast, the first-pass effect was denied a significant item although the first-pass effect was a reputed predictor for the favorable outcome after endovascular thrombectomy. In this study, the first-pass effect was not significantly associated with the early neurological aggravation nor a favorable outcome. In terms of procedural complications, non-intracranial bleeding was not significantly associated with neurological outcomes, whereas procedural intracranial bleeding reduced the probability of favorable outcomes.

Because accurate and standardized performance measurement is crucial in acute stroke management, several scoring systems have been developed to predict clinical outcomes in an acute stroke setting (Supplementary Table 2). The iSCORE has been designed to predict 30-day and 1-year mortalities of patients with acute stroke after hospitalization (17). Although the iSCORE performed well in predicting mortality reflected by high C-statistics, the tool was not appropriate in an acute stroke with reperfusion therapy setting. In contrast, Pittsburgh Outcomes After Stroke Thrombectomy (POST) score targets acute stroke patients within 8 h of being last seen well and undergoing EVT (18). The POST score comprises three items showing high predictability for functional independence at 90 days. However, the POST score has been designed to predict outcomes instead of the procedural performance, and the items included in this score (age, 24–72 h final infarct volume, and hemorrhagic transformation) are substantially unmodifiable outcome predictors. In particular, measurement of the final infarct volume is somewhat difficult because the infarct volume might not be consistent across different imaging modalities and analytical programs (10, 19). Furthermore, the MT-DRAGON is designed to predict functional independence at 90 days using clinical and imaging parameters (20). Specifically, the MT-DRAGON score includes the DWI-ASPECT score instead of infarct volume. Moreover, the MT-DRAGON score has been developed to predict clinical outcomes following thrombectomy. However, the complexity of calculating the score has rendered its prompt use challenging.

Compared with the abovementioned scores, the TAB-TICI score has several distinctive features. The TAB-TICI score is targeted at measuring the successful recanalization rather than predicting the outcome of each patient. The determinants of neurological outcomes associated with thrombectomy can be categorized into unmodifiable factors such as demographics and stroke severity and modifiable factors such as in-hospital workflow time metrics and procedural complications. Because prior scores typically used unmodifiable predictors as items, these scores are inappropriate to evaluate performance (Supplementary Table 2). Contrary to these scores, the TAB-TICI score was developed by exclusively selecting modifiable predictors that are amenable toward improvement. Therefore, the TAB-TICI score seems to be suitable for quality assurance/control, longitudinal monitoring, comparisons among different organizations, and academic activities. Another remarkable peculiarity of the TAB-TICI score is that it is the most straightforward scoring system to remember and calculate, without requiring a calculating chart or an electronic calculator.

Notably, the TAB-TICI score and its derived procedural performance grade, which were used as surrogate markers for procedural performance, were significantly associated with the volume of thrombectomy cases at each site and in each admission year. Although second-generation EVT devices are easy to use, multiple studies have demonstrated the pattern of a learning curve in thrombectomy procedures (21, 22) and the mode of the learning curve differed according to the technique (23). This practitioner-level learning curve effect can be presented as the center-level cumulative volume effect. Kim and colleagues analyzed the impact of accumulated volume of thrombectomy cases at multiple centers and reported that the accumulated volume of thrombectomy cases affected outcomes, safety, and workflow time (7). Decrease in the TAB-TICI score with the later admission years can be interpreted in the same context. Between 2012 and 2017, two critical changes regarding EVT occurred in Korea; in August 2014, retrievable stent reimbursement was approved for the use of Solitaire FR and TREVO in acute stroke patients, and in February 2015, the results of three successful randomized clinical trials were reported, which entailed the updating of clinical practice guidelines to recommend EVT for eligible acute stroke patients (24). These changes could improve the overall performance of EVT, reflected as an increase in the proportion of excellent performance grades.

This study has limitations. First, the study population was entirely Korean, and the validity of this score should be confirmed in different populations. Second, this study included both anterior and posterior circulation stroke patients. However, sensitivity analysis revealed comparable outcome predictions between anterior and posterior circulation stroke patients. Third, the clinical usefulness of the TAB-TICI score should be validated to be accepted as a useful score.

Despite these limitations, the present study provides a novel tool to measure the procedural performance of thrombectomy. Performance of the TAB-TICI score was stable an independent populations and their subgroups, and the excellent, good, and poor performance grades derived from the TAB-TICI score were significantly associated with the surrogate markers for the successful recanalization. Therefore, the TAB-TICIE score, being simple and easy to use, is expected to become a useful tool for measuring and improving thrombectomy performance in acute stroke patients with LVO.

## Data Availability Statement

Anonymized data will be provided upon request to the corresponding author.

## Ethics Statement

The studies involving human participants were reviewed and approved by Samsung Medical Center IRB SMC-2016-08-119. Written informed consent for participation was not required for this study in accordance with the national legislation and the institutional requirements.

## Author Contributions

W-KS performed statistical analysis, provided the tables and figures, and wrote the first draft of the manuscript. HN supervised the project. All authors were involved in the design and interpretation of the data, edited the manuscript, and approved the final draft.

## Funding

This work was partially supported by the National Research Foundation of Korea (NRF) grant (2020M3E5D2A01084891; W-KS) and by a grant of the Korea Health Technology R&D Project through the Korea Health Industry Development Institute (KHIDI), funded by the Ministry of Health and Welfare, Republic of Korea (Grant Numbers: HI19C0481 and HC19C0028).

## Conflict of Interest

W-KS received honoraria for lectures from Pfizer, Sanofi-Aventis, Otsuka Korea, Dong-A Pharmaceutical Co, Ltd, Beyer, Daewoong Pharmaceutical Co, Ltd, Daiichi Sankyo Korea Co, Ltd, and Boryung Pharmaceutical; a study grant from Daiichi Sankyo Korea Co, Ltd; consulting fees from OBELAB Inc.; and a stock option from JLK INSPECTION. The remaining authors declare that the research was conducted in the absence of any commercial or financial relationships that could be construed as a potential conflict of interest.

## Publisher's Note

All claims expressed in this article are solely those of the authors and do not necessarily represent those of their affiliated organizations, or those of the publisher, the editors and the reviewers. Any product that may be evaluated in this article, or claim that may be made by its manufacturer, is not guaranteed or endorsed by the publisher.
