# Supplementary material for: TAB-TICI Score: Successful Recanalization Score After Endovascular Thrombectomy in Acute Stroke
Source: Front Neurol. 2021 Oct 14;12:692490. doi: 10.3389/fneur.2021.692490 (PMC8551570; doi:10.3389/fneur.2021.692490)
Supplement: Supplementary file 1 [file Table_1.DOCX]

**TAB-TICI Score: Successful Recanalization Score After Endovascular Thrombectomy in Acute Stroke**

Subtitle: TAB-TICI score as a successful recanalization

# Contents

# Table I. Selection of Items From the Major Categories Related to Early Neurological Aggravation and Favorable Outcomes

# Table II . Selection of items from the major categories related to early neurological aggravation and favorable outcomes

# Table III. Items and coefficients used in iSCORE, POST, MT-DRAGON, and TAB-TICI scores

# Figure I. Receiver operating curves in *k*-fold validation of the TAB-TICI scores for favorable outcomes and early neurological aggravation

Figure II. Association between the TAB-TICI score and accumulated proportion of early neurological aggravation and favorable outcomes

# **Table I. Selection of Items From the Major Categories Related to Early Neurological Aggravation and Favorable Outcomes**

|  | Early neurological aggravation | | | | Favorable outcome | | | |
| --- | --- | --- | --- | --- | --- | --- | --- | --- |
|  | Univariate |  | Multivariate* |  | Univariate |  | Multivariable^*^ |  |
|  | OR (95% CI) | *P* Value | OR (95% CI) | *P* Value | OR (95% CI) | *P* Value | OR (95% CI) | *P* Value |
| ER-to-puncture >110 min | 1.80 (1.08–3.00) | 0.03 | 1.73 (1.00–2.97) | 0.048 | 0.58 (0.38–0.88) | 0.01 |  |  |
| Puncture-to-recanalization >35 min | 1.43 (0.89–2.28) | 0.14 |  |  | 1.47 (0.99–2.20) | 0.06 |  |  |
| Presence of adjuvant device | 3.35 (2.01–5.59) | <0.01 | 3.40 (1.91–6.04) | <0.01 | 0.36 (0.21–0.62) | <0.01 | 0.49 (0.26–0.90) | 0.02 |
| Adjuvant chemical thrombolysis | 1.64 (1.02–2.63) | 0.04 |  |  | 0.96 (0.65–1.43) | 0.85 |  |  |
| Non-procedural adverse events | 0.82 (0.30–2.19) | 0.69 |  |  | 0.96 (0.44–2.10) | 0.91 |  |  |
| Procedural intracranial bleeding | 2.23 (1.07–4.67) | 0.03 |  |  | 0.19 (0.07–0.51) | 0.01 | 0.24 (0.09–0.66) | 0.01 |
| Failed thrombectomy (mTICI 0–2a) | 2.98 (1.80–4.9) | <0.01 | 2.01 (1.14–3.55) | 0.02 | 0.18 (0.10–0.32) | <0.01 | 0.25 (0.14–0.45) | <0.01 |

*Multivariate logistic model was the final model using backward conditional covariate selection.

# **Table II. ROC curve analysis of comparison among predictive models for early neurological aggravation and favorable outcomes**

| Model | ROC area | Std. Err. | Chi^2^ | Pr > Chi^2^ |
| --- | --- | --- | --- | --- |
| Early neurological aggravation |  |  |  |  |
| Time + Adjuvant device + Procedural bleeding (0–2) + Recanalization (Standard) | 0.7237 | 0.0311 |  |  |
| Time + Adjuvant device (0–2) + Procedural bleeding (0–2) + Recanalization (0–2) | 0.7384 | 0.0307 | 3.7725 | 0.0521 |
| Time + Adjuvant device + Procedural bleeding + Recanalization | 0.7303 | 0.0297 | 0.638 | 0.4244 |
| Time + Adjuvant device (0–2) + Procedural bleeding + Recanalization | 0.7319 | 0.03 | 0.8903 | 0.3454 |
| Time + Adjuvant device (0–2) + Procedural bleeding (0–2) + Recanalization | 0.7245 | 0.0311 | 0.0888 | 0.7657 |
| Time + Adjuvant device + Procedural bleeding + Recanalization (0–2) | 0.7471 | 0.0294 | 4.6724 | 0.0307 |
| Time + Adjuvant device (0–2) + Procedural bleeding + Recanalization (0–2) | 0.7471 | 0.0297 | 4.5666 | 0.0326 |
| Time + Adjuvant device + Procedural bleeding (0–2) + Recanalization (0–2) | 0.7383 | 0.0306 | 3.9103 | 0.048 |
| Poor functional outcome |  |  |  |  |
| Time + Adjuvant device + Procedural bleeding (0–2) + Recanalization (Standard) | 0.7132 | 0.0295 |  |  |
| Time + Adjuvant device (0–2) + Procedural bleeding (0–2) + Recanalization (0–2) | 0.7164 | 0.0293 | 0.5938 | 0.4409 |
| Time + Adjuvant device + Procedural bleeding + Recanalization | 0.7153 | 0.0293 | 0.2266 | 0.6341 |
| Time + Adjuvant device (0–2) + Procedural bleeding + Recanalization | 0.7166 | 0.0292 | 0.51 | 0.4752 |
| Time + Adjuvant device (0–2) + Procedural bleeding (0–2) + Recanalization | 0.7145 | 0.0294 | 0.7072 | 0.4004 |
| Time + Adjuvant device + Procedural bleeding + Recanalization (0–2) | 0.7181 | 0.0291 | 0.6916 | 0.4056 |
| Time + Adjuvant device (0–2) + Procedural bleeding + Recanalization (0–2) | 0.719 | 0.029 | 0.9477 | 0.3303 |
| Time + Adjuvant device + Procedural bleeding (0–2) + Recanalization (0–2) | 0.7154 | 0.0293 | 0.3181 | 0.5727 |

Time, ER-to-puncture time >110 min; Adjuvant device (0–2) was rated as none (0), one class of adjuvant device use (1), and two or more classes of adjuvant device use (2); Procedural bleeding (0–2) was rated as none (0), ICH only (1), associated SAH or IVH (2); Recanalization (0–2) was rated as mTICI 2b-3 (0), mTICI 2a (1), and mTICI 0-1 (2).

# **Table III. Items and coefficients used in iSCORE, POST, MT-DRAGON, and TAB-TICI scores**

| Title | Year | Target population | Aim | Items included in each score | | Score range |
| --- | --- | --- | --- | --- | --- | --- |
|  |  |  |  | Modifiable predictors | Unmodifiable predictors |  |
| iSCORE^18^ | 2011 | Acute stroke after hospitalization | To predict 30-day or 1-year mortality |  | Age, sex, stroke severity, stroke subtype, risk factor, comorbid condition, preadmission disability, and glucose at admission | 0–285 |
| POST^19^ | 2014 | Anterior circulation large vessel occlusion with endovascular therapy within 8 h from LSW | To predict mRS 0–2 at 90 days |  | Age, final infarct volume, and parenchymal hematoma type 1 or 2 | 0–120 |
| MT-DRAGON^21^ | 2019 | Anterior circulation large vessel occlusion with endovascular therapy within 8 h from LSW | To predict mRS 0–2 at 90 days | Onset-to-groin puncture time >4.5 h | DWI-ASPECT ≤ 4.5 h, pre-stroke mRS >1, age, glucose, occlusion site, and NIHSS | 0–20 |
| TAB-TICI | 2019 | Acute stroke with large vessel occlusion with endovascular therapy within 24 h from LSW | To measure thrombectomy procedural performance | ER-to-puncture time <110 min, adjuvant thrombectomy device use, procedural bleeding, and mTICI |  | 0–5 |

# **Figure I.** Receiver operating curves in *k*-fold validation of the TAB-TICI score for favorable outcomes and early neurological aggravation


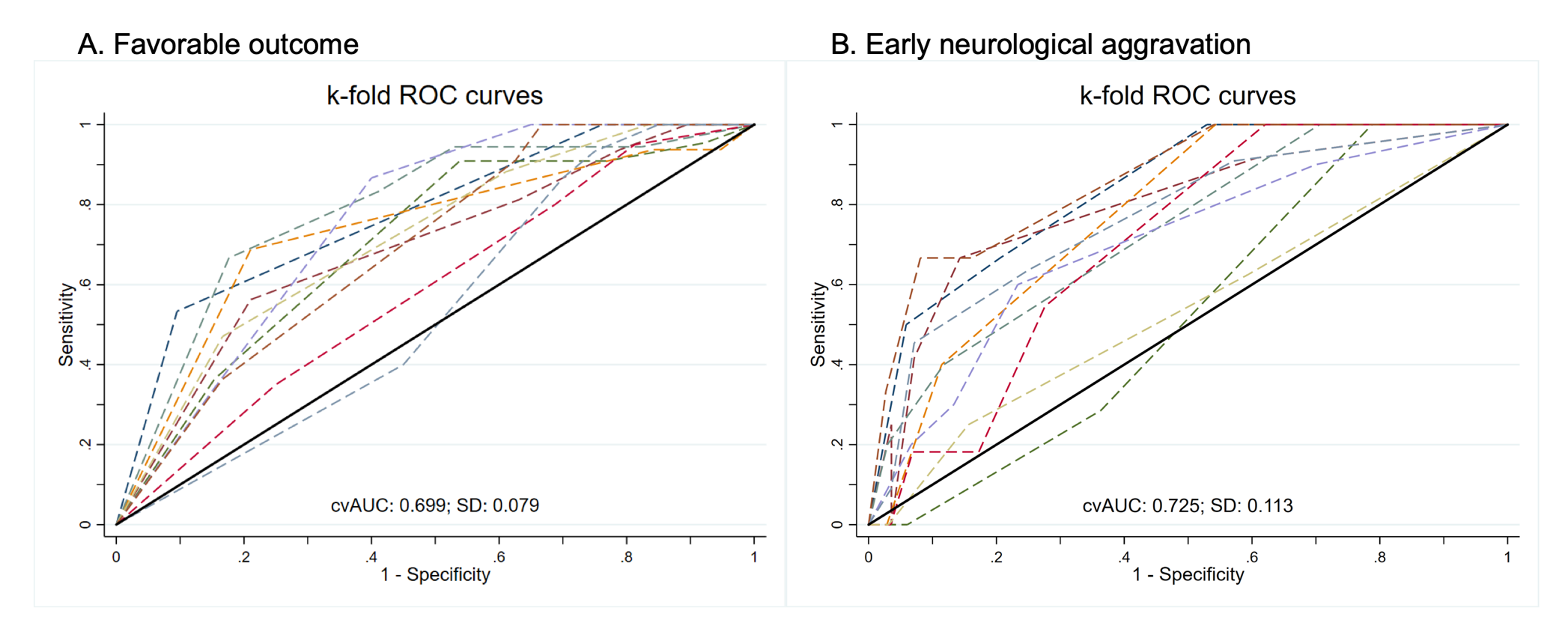


# **Figure II.** Association between the TAB-TICI score and accumulated proportion of early neurological aggravation and favorable outcomes


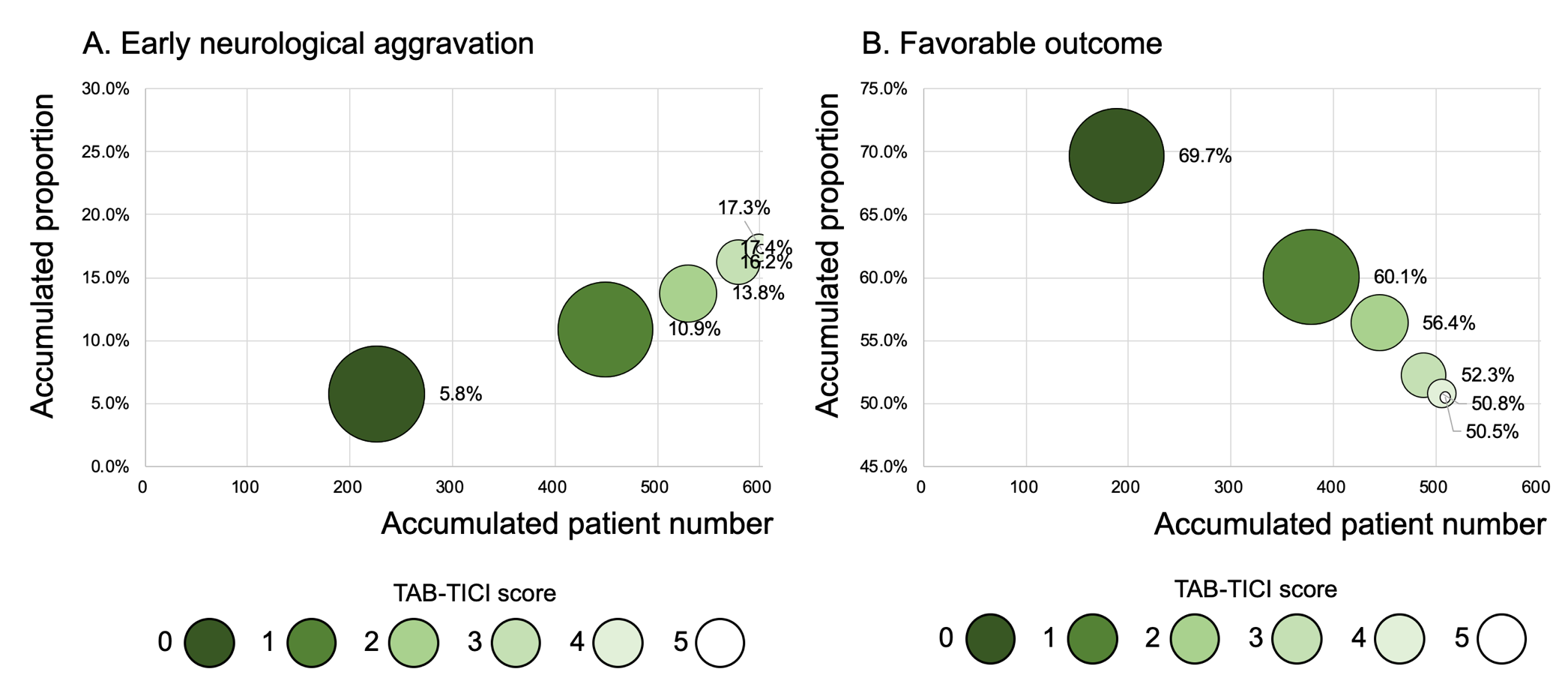


The radius of the circle represents the number of the patients with that score.

The accumulated proportion of early neurological aggravation distributed according to the TAB-TICI score: <10% (TAB-TICI score 0), 10%–15% (TAB-TICI score 1-2), and >15% (TAB-TICI score 3–5). The accumulated proportion of favorable outcomes distributed according to the TAB-TICI score: 50%–55% (TAB-TICI score 3–5), 5%–60% (TAB-TICI score 2), and >60% (TAB-TICI score 0-1).
